# Genomic Features and Antimicrobial Susceptibility of *Listeria innocua* Isolated from Raw Drinking Milk in Poland

**DOI:** 10.3390/foods15061017

**Published:** 2026-03-13

**Authors:** Pierre-Emmanuel Douarre, Renata Pyz-Łukasik, Grzegorz Borsuk, Waldemar Paszkiewicz

**Affiliations:** 1 Salmonella and Listeria Unit, Laboratory for Food Safety, ANSES, 94700 Maisons-Alfort, France; pierre-emmanuel.douarre@anses.fr; 2 Department of Food Hygiene of Animal Origin, Faculty of Veterinary Medicine, University of Life Sciences in Lublin, Akademicka 12, 20-033 Lublin, Poland; waldemar.paszkiewicz@up.edu.pl; 3 Institute of Biological Bases of Animal Production, University of Life Sciences in Lublin, Akademicka 13, 20-950 Lublin, Poland; grzegorz.borsuk@up.edu.pl

**Keywords:** *Listeria innocua*, raw drinking milk, whole genome sequencing, virulence genes, resistance genes, antimicrobial susceptibility

## Abstract

*Listeria innocua* is a bacterium frequently detected in food and food production plants (FPPs). Understanding the heterogeneity of *L. innocua* food isolates is essential for predicting potential food safety threats and developing preventive and control measures. This study aimed to characterize *L. innocua* isolated from raw drinking milk by investigating the genomic features related to virulence, antimicrobial resistance, and persistence using whole-genome sequencing (WGS), along with phenotypic antimicrobial susceptibility testing using the disk diffusion method. All ten isolates analyzed in this study belonged to sequence type (ST) 492 and were distantly related to the reference strain. A total of 80 virulence-associated genes were identified, including the complete *Listeria* Pathogenicity Islands-3 (LIPI-3) and LIPI-4 clusters typically found in virulent *L. monocytogenes* clones, as well as 66 additional genes involved in adhesion, invasion, motility, post-translational modification, regulation, immune modulation, and stress survival. Stress survival islet 2 (SSI-2) and genes encoding the *Clp* protease complex (*clpC*, *clpE*, *clpP*), which support both persistence and virulence, were also detected, whereas LIPI-1 and internalin genes were not detected. The antimicrobial resistance determinants included *fosX*, *lin*, *norB*, *sul*, and three multidrug efflux pumps (*lde*, *mdrL* and *mdrM*). Mobile genetic elements (plasmids, prophages, or transposons) were not detected. All isolates were phenotypically susceptible to benzylpenicillin, ampicillin, meropenem, erythromycin, and trimethoprim–sulfamethoxazole. These findings underscore the importance of ongoing genomic surveillance of *L. innocua* in food environments and highlight the need to assess the potential risk posed by specific lineages, such as ST492, to food safety.

## 1. Introduction

*Listeria innocua* is a ubiquitous, Gram-positive, facultative anaerobic, non-spore-forming bacterium classified as a non-pathogenic species of the genus *Listeria*. This bacterium is a common contaminant of FPPs [1,2,3,4]. Eliminating *L. innocua* from FPP is difficult even after applying standard cleaning and disinfection procedures, thereby increasing the risk of food contamination [5]. Although *L. innocua* is considered an indicator of poor hygiene in FPP, food laws do not define a criterion for the acceptable functioning of the production process [2,6].

*L. innocua* shares a close evolutionary relationship with *L. monocytogenes*. *L. monocytogenes* is one of the most dangerous foodborne pathogens for which food safety criteria have been established [6]. Both species evolved from a common virulent ancestor, with their primary differences arising from the loss of key virulence-associated genes in *L. innocua* [7]. Nevertheless, some *L. innocua* strains retain functional homologues of *L. monocytogenes* virulence genes [7,8], as evidenced by reports of serious human infections, including fatalbacteremia, meningitis, ventriculoperitoneal drainage infections, and neonatal listeriosis [9,10,11,12,13]. Moreover, *L. innocua* is capable of acquiring virulence and resistance genes, and also sharing them with *L. monocytogenes*, with which it often coexists in the same environmental niches [14,15,16,17], which may have implications for food safety. Transmission of *Listeria* infections to humans is primarily associated with ready-to-eat (RTE) food [18]. The health risk associated with RTE food depends mainly on the effectiveness of preventive and control measures implemented by food business operators. Understanding the genetic variability of *L. innocua* among food isolates is important for predicting potential food safety threats and developing effective preventive and control measures [19].

Antimicrobial resistance, including antibiotic resistance, poses a significant challenge to global public health [20]. Monitoring resistance at regional, national, and international levels is relevant for public health, as it provides early warning of emerging threats and identifies long-term trends in resistance [21]. Antibiotic resistance phenotypic testing has long been considered the gold standard in clinical applications, providing the basis for clinical diagnosis of antibiotic resistance and treatment optimization [22]. Antibiotic treatment for *Listeria* infections, depending on the clinical case, involves penicillin, ampicillin, trimethoprim-sulfamethoxazole, meropenem, and erythromycin [23]. The phenotypic resistance of *L. innocua* to these and other antimicrobials has been described in the literature [3,24,25], emphasizing the need for continuous surveillance.

The present study aimed to characterize the genomic features that contribute to virulence, antimicrobial resistance, and persistence in FPPs, as well as the phenotypic antimicrobial susceptibility of *Listeria innocua* isolated from raw drinking milk in Poland.

## 2. Materials and Methods

### 2.1. Strain Isolation and DNA Extraction

*L. innocua* isolates (*n* = 10) were recovered in 2023 from raw drinking milk produced on an organic farm in Eastern Poland. Each isolate originated from a different production batch, with one bottle of milk (original, single-use, and sealed packaging) representing one production batch. A total of 23 production batches were analyzed in this study. The milk was delivered to a retail store on a weekly cycle (once a week on the same day). The study continued for several subsequent weeks, during which *L. innocua* was detected intermittently. Raw drinking milk was purchased from a retail store and transported to the laboratory in its original packaging at 0–4 °C. According to EU legislation, raw drinking milk is classified as RTE food [6,26]. *L. innocua* was isolated in accordance with the International Standard PN-EN ISO 11290-1:2017-07 [27] using microbiological media from Biomaxima, Lublin, Poland. DNA was isolated from pure bacterial cultures grown on agarose medium plates using a Genomic Micro AX Bacteria+ DNA kit (A&A Biotechnology, Gdańsk, Poland). The obtained DNA was suspended in Tris buffer (10 mM, pH 8.5). The concentration of genomic DNA was measured prior to library preparation using fluorimetry with the Quant-iT™ PicoGreen™ dsDNA Assay Kit reagent (Life Technologies, Carlsbad, CA, USA). Measurements were performed using an Infinite instrument (Tecan, Männedorf, Switzerland).

### 2.2. Library Preparation and Sequencing

Genomic DNA was fragmented by sonication using a Covaris E210 apparatus (Covaris, Woburn, MA, USA), according to the recommended parameters for preparing libraries for Illumina sequencing. Libraries were prepared using the NEBNext^®^ Ultra™ II DNA Library Prep Kit for Illumina^®^ (New England Biolabs, Ipswich, MA, USA) according to the manufacturer’s instructions. Quality control of the libraries was performed using a Bioanalyzer (Agilent, Santa Clara, CA, USA) and quantitative PCR (qPCR). High-throughput sequencing was performed using a MiSeq sequencer (Illumina, San Diego, CA, USA) with 2 × 300 nt paired-end technology and a MiSeq Reagent Kit v3 600-cycle (Illumina, San Diego, CA, USA), following the manufacturer’s protocol. Adapter sequences and low-quality bases were removed using Fastp v0.23.4. Read quality was assessed using FastQC v0.12.1 and summarized using MultiQC v1.9.

### 2.3. Genomic Analysis

De novo assembly from short reads was performed using Spades (v3.15.5), and genomes were annotated using Prokka (v1.14.6) [28,29]. The quality of the genome assemblies was assessed using QUAST v5.3.0 [30]. The sequence type (ST) was determined based on the Listeria Multilocus Sequence Typing (MLST) scheme from Institut Pasteur [31].

Variant calling to identify mutations was performed using the bacterial SNP-calling pipeline Snippy (v4.6.0) with the default parameters (https://github.com/tseemann/snippy) (accessed on 7 March 2026). The reads from the *L. innocua* isolates were aligned against the ATCC 33090 reference genome (CP117229.1). To assess the genetic relationships among the ten strains, the assembly of isolate LI54 was used as a reference and reads from the remaining nine isolates were mapped to it.

Pangenome analysis was conducted using Panaroo (v1.2.7) in strict mode [32]. Genomad (v1.8.0) was run with default parameters to identify putative mobile genetic elements, while PlasmidFinder and PHASTEST (https://phastest.ca/) (accessed on 7 March 2026) were used to detect the presence or absence of plasmid and phage regions, respectively [33,34,35]. Virulence genes from the VFDB database [36] were screened across *L. innocua* genomes, and AMRFinderPlus (v3.10.18) was used to identify antimicrobial resistance genes [37]. Additionally, heavy metal and biocide resistance genes were screened using the BacMet database [38] with DIAMOND (v0.9.24) blastp (https://github.com/bbuchfink/diamond) (accessed on 7 March 2026). To confirm these predictions, sequencing reads or assembled genomes were further analyzed by mapping or BLAST-based (v2.16.0) searches against the virulence and resistance gene collection available in the Listeria BIGSdb (https://bigsdb.pasteur.fr/listeria/) (accessed on 7 March 2026). Similarly, all loci of *Listeria* pathogenicity islands (LIPIs), *Listeria* Genomic Islands (LGIs) and Stress Islands (SSI-1 and 2) from BIGSdb were searched in the *L. innocua* genomes.

To compare the isolates analyzed in this study within the broader *L. innocua* population, 1577 publicly available genome assemblies and associated metadata were retrieved from the NCBI database. Multilocus sequence typing (MLST) was performed on all genomes, and isolates were categorized by source as animal, food, environment, or human. A minimum spanning tree (MST) was constructed using GrapeTree v2.1 to compare the ten isolates from this study with those from the NCBI database [39]. Accession numbers, metadata, and MLST results are listed in Supplementary Table S1. To investigate the phylogenetic relationship between the isolates in this study and ten other ST492 isolates, sequencing reads were mapped to the LI54 genome to generate a core alignment, and recombination regions were identified and removed using Gubbins v2.4.1 [40]. The resulting recombination-free core alignment was used to construct a maximum-likelihood tree using IQ-TREE v2.1.2 [41]. The pangenome of the additional ST492 was examined as described above. Tree visualization and annotation were performed using iTOL v7.0 [42].

### 2.4. Antimicrobial Susceptibility

The susceptibility of the isolates to the five antibiotics was determined using the disk diffusion method. An inoculum with a density of 0.5 McFarland scale of each isolate was plated on Mueller-Hinton agar with a 5% addition of defibrinated horse blood and 20 mg/L β-NAD (MH-F) (Biomaxima, Lublin, Polska), and discs with benzylpenicillin (1 U), ampicillin (2 µg), meropenem (10 µg), erythromycin (15 µg), and trimethoprim-sulfamethoxazole (1.25–23.75 µg) were then added (Biomaxima, Lublin, Polska). The antibiograms were incubated in an atmosphere enriched with 5% CO_2_ at 35 °C for 18 h ±2 h. The growth inhibition zones around the antibiotic discs were measured and analyzed in accordance with the European Committee on Antimicrobial Susceptibility Testing (EUCAST) v. 12.0 [43]. The ATCC 49619 strain of *Streptococcus pneumoniae* was used for quality control.

## 3. Results

### 3.1. Sequencing and Quality Control

The read and assembly quality metrics are summarized in Supplementary Table S2. Each sample consisted of paired-end reads (R1 and R2), with read counts ranging from approximately 571,000 to 834,000 reads per file. The average read lengths after trimming ranged from 241 bp to 269 bp, and the GC content was consistent across all samples at 37%, except for LI65, which showed a slightly higher GC content of 38%. All read files passed the key FastQC quality modules, indicating high-quality reads suitable for performing de novo assembly. All assemblies demonstrated high quality, with total genome lengths of 2.79 Mb, consistent with the expected genome size for *L. innocua*. The number of contigs ≥1000 bp ranged from 7 to 10, with most assemblies showing a high degree of continuity (N50 values between 468 kb and 1.44 Mb).

### 3.2. MLST and Core Genome SNP Analysis

The allelic profiles of the seven housekeeping genes were determined as follows: *abcZ* 36, *bglA* 21, *cat* 40, *dapE* 45, *dat* 48, *ldh* 179, and *lhkA* 61, allowing the assignment of all the analyzed *L. innocua* isolates to the same sequence type, ST492. Single-nucleotide polymorphism (SNP) analysis revealed that all analyzed isolates were virtually identical, differing by only 4–6 SNPs, while they were distantly related to the reference strain *L. innocua* ATCC 33090 (28,003–28,149 SNPs difference), highlighting the broader genomic diversity of this species (Supplementary Table S3).

### 3.3. Pangenome Analysis, Virulence and Resistance Factor Screening

Pangenome analysis showed no differences in gene content (2727 genes), confirming that these isolates represent the same strain or a very recent clonal variant. Screening for virulence factors, antimicrobial resistance genes, and stress response markers yielded identical results across all isolates (Table 1). A total of 80 virulence factors were identified, spanning functional categories such as adherence, exotoxins, immune modulation, invasion, motility, post-translational modification, regulation, and stress survival. Notably, the isolates harbored all the genes of the LIPI-3 (*llsA*, *llsG*, *llsH*, *llsX*, *llsB*, *llsY*, *llsD*, and *llsP*) and LIPI-4 (genes 70009–70014 in LM9005581). Additionally, 29 genes involved in the biosynthesis and regulation of flagellar proteins and 12 genes implicated in the regulation of virulence factors were identified. Five genes playing a significant role in bacterial fitness under environmental stress conditions, including two genes from the SSI-2 and three genes from the *Clp* protease complex were also identified, whereas the five genes of SSI-1 were absent. Overall, ten *L. innocua* isolates encoded most of the virulence factors commonly present in *L. monocytogenes*, whereas specific virulence genes from LIPI-1 (*prfA, hly, mpl, actA, plcB*, and *plcA*) and 18 internalin genes were absent in *L. innocua*. The antimicrobial resistance genes *fosX*, *lin*, *norB*, and *sul* were detected, along with three multidrug efflux pumps: *lde*, *mdrL* and *mdrM*. Furthermore, *Listeria* Genomic Islands 1 (LGI1), LGI2, and LGI3 were not detected in the *L. innocua* isolates. Finally, no mobile genetic elements, such as plasmids, prophages, or transposons were identified in any of the analyzed genomes.

### 3.4. Listeria innocua Population and ST492 Isolates

**Figure 1:**
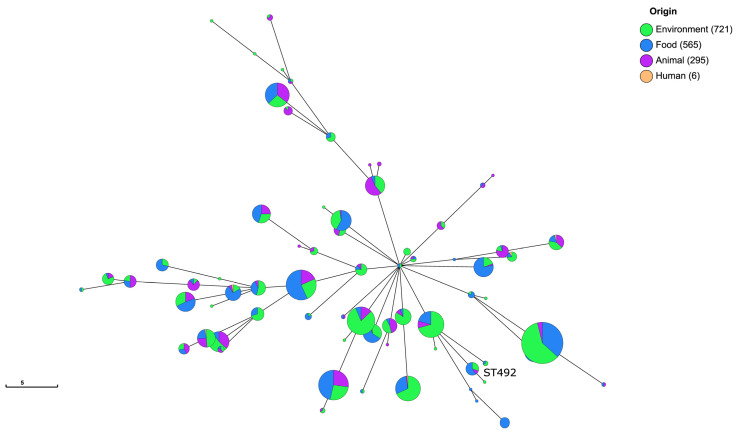
Minimum spanning tree (MST) analysis based on the MLST allelic profiles of 1587 *L. innocua* isolates. Each circle corresponds to an individual sequence type (ST) and its size is proportional to the number of isolates within that ST. Circle colors indicate the source of the isolates (environment, food, animal and human). Connecting lines represent allelic difference between STs.

MLST-based minimum spanning tree (MST) analysis showed that the ten food isolates from this study clustered with ten publicly available ST492 genomes (Figure 1).

**Figure 2:**
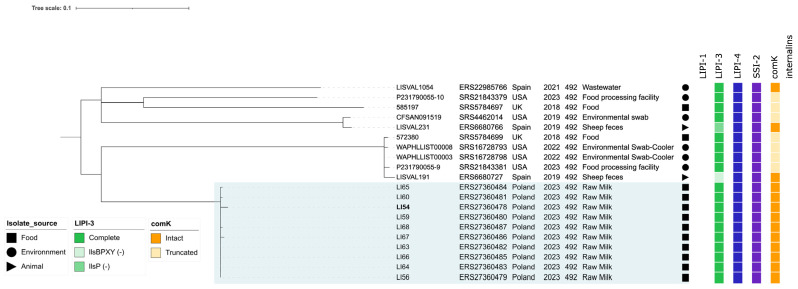
Core-genome SNP-based phylogeny illustrating the evolutionary relationships among *L. innocua* ST492 isolates. Branch lengths are proportional to the number of substitutions per site, and the scale bar indicates the number of substitutions per site. The phylogenetic tree was annotated with geographic location, date of isolation, source, and the presence or absence of selected genes and genomic islands. (-) indicates that the corresponding LIPI-3 genes are absent.

These included isolates recovered from animals (*n* = 2), food (*n* = 2), and environmental sources (*n* = 6) originating from the United Kingdom (*n* = 2), Spain (*n* = 3), and the United States (*n* = 5). A comparison of the seven housekeeping loci further revealed that ST492 is closely related (sharing six of the seven alleles) to ST1417, represented by a single strain isolated from a food-processing environment in Norway, and more distantly related (sharing three of the seven alleles) to ST603 (*n* = 82), which encompasses isolates from diverse sources. Core-genome SNP analysis confirmed the clonal nature of the ten food isolates from this study and their close relationship with other ST492 genomes, while also illustrating the broader genomic diversity within the sequence type, with pairwise SNP distances ranging from 179 to 205 relative to the isolate LI54 (Figure 2).

The overall gene content and virulence factor profiles were highly conserved among the ST492 genomes, with only minor variation observed in the LIPI-3 region and the detection of a truncated *comK* gene, suggesting a potential prophage integration site.

### 3.5. Phenotypic Features of L. innocua Isolates

All isolates were susceptible to benzylpenicillin (1 U), ampicillin (2 µg), meropenem (10 µg), erythromycin (15 µg), and trimethoprim-sulfamethoxazole (1.25/23.75 µg). The zone diameters of all antimicrobial agents exceeded the EUCAST susceptibility breakpoint. The inhibition zone diameters per antibiotic are provided in Table S4.

## 4. Discussion

Understanding the heterogeneity of *L. innocua* among food isolates is important for predicting potential threats to food safety and developing preventive and control measures. This study presents the genomic features of *L. innocua* food isolates, including those contributing to virulence, antimicrobial resistance, and stress survival, as well as their phenotypic responses to clinically relevant antimicrobials in *Listeria* infections.

MLST-based analysis showed that all ten isolates from this study belonged to sequence type ST492, whose genotypic and phenotypic features have not been described previously, highlighting the value of this study. In the comparative MLST dataset used in the study, ST492 was found in only 10 of 1577 public genomes (0.6%); however, these isolates were recovered from food, animals, and environmental sources across three different countries, indicating its potential to adapt and spread across diverse niches. A recent study by Wei et al. [4] also identified two ST492 isolates (4%) in the Ethiopian dairy supply chain, supporting the idea that ST492 may be more widespread than currently documented.

Previous MLST studies have suggested that certain STs may have a specific geographical origin or source. For example, STs such as ST1489, ST1619, ST603, ST537, ST1010, ST3186, ST492, and ST3007 were each restricted to a single region in Ethiopia, whereas ST1087 was found in at least three regions [4]. Similarly, source-based enrichment has been reported, with ST637 and ST1482 overrepresented in agricultural environments, ST537 in slaughterhouses, ST448, ST637, and ST1085 in retail food, and ST1597 and ST603 in dairy facilities [2,44]. However, despite these valuable observations, the distribution and factors shaping the *L. innocua* population structure in different environments remain poorly understood and require further research.

SNP analysis revealed that the ten *L. innocua* isolates from raw drinking milk were highly clonal, differing by fewer than 10 core SNPs. Such a small variation is comparable to that observed among *L. monocytogenes* clones and suggests either the persistence of the same strain within the environment or cross-contamination from a common source. In contrast, a comparison with publicly available ST492 genomes showed 179–205 SNP differences, indicating that this sequence type comprises several closely related clones circulating across various sources and countries. The substantial divergence from the reference strain ATCC 33090 (~28,000 SNPs) further highlights the high genomic heterogeneity of *L. innocua* and indicates the value of including this species in the genomic surveillance of food production environments.

The internalins *inlA* and *inlB*, along with the LIPI-1 cluster (*prfA*, *hly*, *mpl*, *actA*, *plcB*, and *plcA*), are essential for *L. monocytogenes* pathogenesis, mediating adhesion, invasion, intracellular replication, and cell-to-cell spread [45]. A comprehensive study of *L. innocua* confirmed the presence of LIPI-1 and *inlA* (but not *inlB*), including their functionality in both in vitro and in vivo experiments. This study demonstrated *L. innocua*’s ability to actively cross the intestinal epithelium and spread systemically to the liver and spleen, although to a lesser extent than the reference *L. monocytogenes* EGDe strain [8]. None of these genes were confirmed in the analyzed isolates, which is consistent with the results of several other studies [19,44,46]. However, given that *L. innocua* can cause invasive listeriosis in humans and animals in the absence of key virulence genes of *L. monocytogenes* [10,47,48], the results of the present study do not indicate a zero risk of infection, suggesting that other virulence factors should not be excluded.

The eight-gene LIPI-3 (*llsAGHXBYDP*) has been identified exclusively in hypervirulent *L. monocytogenes* strains that encode the bacteriocin listeriolysin S (LLS), which modifies the gut microbiota, enabling efficient colonization of the gut and invasion of deeper organs [45,49]. In *L. innocua*, LIPI-3 followed a reductive evolutionary path towards degeneration, as evidenced by its absence in many strains or signs of decay [8,50,51,52]. Although the presence of LIPI-3 in *L. innocua* was unexpected [50], the analyzed isolates possessed all markers of this virulence cluster. These findings are consistent with those of several other studies that have shown that the full set of LIPI-3 can be present in some *L. innocua* strains, regardless of geographic region, source, or isolation period [8,44,52]. The functionality of LIPI-3 in *L. innocua* has been previously confirmed in vitro [50]. However, the distribution of LIPI-3 within the species and its role in the ecology and adaptation of *L. innocua* remain unexplored [52].

LIPI-4 is a recently identified gene cluster in *L. monocytogenes* that consists of six genes (70,009–70,014) described as cellobiose-family phosphotransferase (PTS) [53]. The distribution of LIPI-4 in *L. monocytogenes* is significantly associated with high virulence of the strains, including those responsible for central nervous system and maternal-neonatal infections [53]. In the present study, LIPI-4 was confirmed in all isolates. These results align with those of other studies, which have demonstrated the widespread occurrence of LIPI-4 among *L. innocua* strains regardless of geographic region, source, or isolation period [8,17,52]. However, the role and function of LIPI-4 in this species remain unknown.

Additionally, genes responsible for secondary or additional virulence factors of *L. monocytogenes* were confirmed in the analyzed isolates, including those related to adhesion (*fbpA*, *lap*), invasion (*iap*/*cwhA*, *gtcA*, and *lpeA*), surface protein anchoring (*lspA*), peptidoglycan modification (*oatA*, *pdgA*), intracellular survival (*lplA1*, *prsA2*), and heat shock proteins (*clpC*, *clpE*, and *clpP*). These results were previously reported in a study on *L. innocua* isolated from the same food matrix (raw milk) [19]. However, the role of these virulence factors in *L. innocua* remains unexplored.

SSI-1 and SSI-2 contribute to the survival of *L. monocytogenes* in suboptimal environmental conditions encountered by this bacterium, both in food and the gastrointestinal tract after ingestion [54]. A prevalence study of their occurrence in *L. monocytogenes* revealed a different distribution of both islands between clinical and food-associated isolates. SSI-1 is widespread in both clinical and food isolates, whereas SSI-2 is primarily found in food isolates and is associated with the persistence of clonally related groups such as ST121 [54]. Of these markers, only SSI-2 was observed in all analyzed *L. innocua* isolates, suggesting a better adaptation to alkaline and oxidative stress conditions, which are often present in FPPs [54]. These results are consistent with those of other studies, which also showed the presence of SSI-2 in *L. innocua* isolated from different environments and the absence of SSI-1 in this species, regardless of the source (food, soil, agricultural water, or FPP) [2,46,55,56]. However, SSI-2 is not a conserved feature of the *L. innocua* genome, as its presence varies even among isolates originating from the same source. These findings indicate the need to determine the role of SSI-2 genes in the environmental fitness of *L. innocua* and to elucidate the importance of SSI-2 heterogeneity in this species.

Monitoring the antimicrobial susceptibility of *Listeria* spp. in both clinical settings and the food chain allows us to verify the effectiveness of current treatments and ensure public health protection [57]. Phenotypic susceptibility to benzylpenicillin, ampicillin, meropenem, erythromycin, and trimethoprim–sulfamethoxazole remains significant for successful clinical management and prevention of complications in listeriosis [23]. In the present study, all *L. innocua* isolates were found to be susceptible to these antimicrobials, indicating that these strains do not pose a concern for current listeriosis treatment strategies. However, previous studies have reported variable susceptibility and occasional resistance to these agents [3,24,25], suggesting that differences in source, geographic region, and isolation period may influence the susceptibility profiles of *L. innocua* populations. Reports on multidrug-resistant *L. innocua,* particularly among food isolates, further support the importance of large-scale surveillance across diverse food matrices [24,57].

In this study, *L. innocua* isolates exhibited an identical genotypic multidrug resistance pattern (*fosX*–*lin*–*norB*–*sul*), predicting resistance to fosfomycin (*fosX*), lincomycin (*lin*), fluoroquinolones, nalidixic acid (*norB*), and sulfonamides (*sul*) [58]. In contrast, Gana et al. [44] reported the presence of 13 antimicrobial resistance (AMR) genes among 110 *L. innocua* isolates, including *tet(M)*, *dfrG*, *mphB*, and *mefA*, with patterns differing across isolates from cattle farms, abattoirs, and retail outlets. These findings illustrate that while *L. innocua* generally harbors only intrinsic resistance determinants [19], certain lineages may acquire additional AMR genes depending on their ecological and geographic context. Together, these results highlight the importance of distinguishing between intrinsic and acquired resistance mechanisms and indicate the importance of ongoing genomic surveillance to monitor the potential emergence and dissemination of acquired resistance in *L. innocua* from food and production environments.

In addition, three efflux pump genes (*lde*, *mdrL* and *mdrM*) were detected in all analyzed isolates. These transporters are known to contribute to the intrinsic multidrug resistance of *L. monocytogenes*, providing tolerance to various antibiotics and disinfectants commonly used in food production environments [59]. *mdrM* also plays a role in intracellular survival by exporting cyclic di-AMP, which triggers type I interferon responses and promotes the virulence of *L. monocytogenes* [59]. The presence of these genes in *L. innocua* is notable, as they have been sporadically reported only in isolates from various sources and geographic regions [19,44,46]. However, their functional relevance in *L. innocua* remains to be elucidated. Further studies are needed to determine whether these efflux systems are active and to what extent they contribute to environmental adaptation and tolerance to disinfectants in food production settings.

Unlike other *L. innocua* strains, ST492 lacks LIPI-1, LGI-1, LGI-2, LGI-3, internalin genes, and mobile genetic elements such as plasmids, prophages, and transposons, which contribute to virulence and antimicrobial resistance in *L. monocytogenes* [16,19,46].

## 5. Conclusions

This study expands the current knowledge on the genomic diversity of *Listeria innocua* by providing an in-depth characterization of lineage ST492, a sequence type detected across multiple sources and geographic regions. The genomic features of ST492 suggest its potential for virulence, multidrug resistance, and adaptation to stress conditions. Although the epidemiological scope of the study remains limited, the close genetic relatedness of the isolates is consistent with but does not confirm intermittent cross-contamination from a common source within the milk production environment. Although the analyzed isolates remained susceptible to clinically relevant antibiotics, the presence of stress- and resistance-associated determinants highlights the importance of monitoring *L. innocua* as part of routine food safety surveillance. Importantly, while several determinants homologous to virulence factors described in *L. monocytogenes* have been identified, their roles and functional relevance in *L. innocua* remain to be elucidated. Future studies integrating genomic, transcriptomic, and phenotypic approaches will be useful for assessing the functional relevance of these determinants and better understanding the ecological and public health significance of emerging *L. innocua* lineages.

## Figures and Tables

**Table 1:** Virulence and resistance markers identified in the *L. innocua* isolates.

	Functional Category	Occurrence	Genes	DATABASE
Virulence (*n* = 80)	Adherence	2	*fbpA*, *lap*	BIGSdb & VFDB
	Exotoxin	8	LIPI-3 (*llsA*, *llsB*, *llsD*, *llsG*, *llsH*, *llsP*, *llsX*, *llsY*)	BIGSdb & VFDB
	Immune modulation	2	*oatA*, *pdgA*	BIGSdb & VFDB
	Invasion	8	*cwhA*, LIPI-4 (LM9005581_70009-70014), *lpeA*	BIGSdb & VFDB
	Motility	29	*cheR*, *cheV*, *flaA*, *flgB*, *flgC*, *flgD*, *flgE*, *flgG*, *flgK*, *flgL*, *flhA*, *flhB*, *flhF*, *fliD*, *fliE*, *fliF*, *fliG*, *fliH*, *fliI*, *fliM*, *fliN*, *fliN*, *fliP*, *fliQ*, *fliR*, *fliS*, *fliY*, *motA*, *motB*	BIGSdb & VFDB
	Nutritional/Metabolic factor	6	*gltA*, *gltB*, *lplA1*, *oppA*, *purQ*, *svpA*	BIGSdb & VFDB
	Post-translational modification	8	*dltA*, *gtcA*, *lgt*, *lspA*, *prsA2*, *srtA*, *srtB*, *stp*	BIGSdb & VFDB
	Regulation	12	*agrA*, *agrC*, *cheA*, *cheY*, *comK*, *fur*, *lisK*, *lisR*, *codY*, *pdeE*, *virR*, *virS*	BIGSdb & VFDB
	Stress survival	3	*clpC*, *clpE*, *clpP*	VFDB
	Stress survival	2	SSI-2 (lin0464 and lin0465)	BIGSdb
Resistance (*n* = 7)	Antimicrobial resistance	1	*fosX*	BIGSdb &AMRFinderPlus
	Antimicrobial resistance	3	*lin*, *norB*, *sul*	BIGSdb
	Multidrug resistance	1	*mdrM*	BIGSdb
	Multidrug resistance	2	*mdrL* and *lde*	Bacmet

## Data Availability

The data presented in this study are openly available in the European Nucleotide Archive (ENA) under the accession PRJEB104025 (secondary accession: ERP185338). Data repository: https://www.ebi.ac.uk/ena/browser/view/PRJEB104025 (accessed on 7 March 2026). Individual accession numbers are listed in Supplementary Table S2.

## Supplementary Materials

The following supporting information can be downloaded at: https://www.mdpi.com/article/10.3390/foods15061017/s1; Table S1: Metadata and MLST results of publicly available *Listeria innocua* genomes; Table S2: Sequencing reads and assembly quality metrics for *Listeria innocua* isolates; Table S3: Core-genome SNP differences identified by Snippy among the ten *Listeria innocua* isolates and reference genomes; Table S4: Inhibition zone diameters per antibiotic.

## Abbreviations

The following abbreviations are used in this manuscript:

EUCAST	The European Committee on Antimicrobial Susceptibility Testing
MST	Minimum Spanning Tree
SNP	Single-Nucleotide Polymorphism
LIPIs	*Listeria* Pathogenicity Islands
LGIs	*Listeria* Genomic Islands
SSI	Stress Survival Islet
MLST	Multilocus Sequence Typing
FPPs	Food Production Plants
RTE food	Ready-to-Eat Food
WGS	Whole-Genome Sequencing
ST	Sequence Type
